# Identification of Entry Inhibitors against Delta and Omicron Variants of SARS-CoV-2

**DOI:** 10.3390/ijms23074050

**Published:** 2022-04-06

**Authors:** Richard Kuan-Lin Lee, Tian-Neng Li, Sui-Yuan Chang, Tai-Ling Chao, Chun-Hsien Kuo, Max Yu-Chen Pan, Yu-Ting Chiou, Kuan-Ju Liao, Yi Yang, Yi-Hsuan Wu, Chen-Hao Huang, Hsueh-Fen Juan, Hsing-Pang Hsieh, Lily Hui-Ching Wang

**Affiliations:** 1 Institute of Molecular and Cellular Biology, College of Life Science, National Tsing Hua University, Hsinchu 300013, Taiwan; richard@smobio.com (R.K.-L.L.); tiannengl@gmail.com (T.-N.L.); maxsos1996@hotmail.com (M.Y.-C.P.); chaos5352@gmail.com (Y.-T.C.); joeyyang@gapp.nthu.edu.tw (Y.Y.); e8010281@gmail.com (Y.-H.W.); 2 SMOBIO Technology, Inc., Hsinchu 300096, Taiwan; kuo@smobio.com; 3 Department of Clinical Laboratory Sciences and Medical Biotechnology, College of Medicine, National Taiwan University, Taipei 100225, Taiwan; sychang@ntu.edu.tw (S.-Y.C.); d01424001@ntu.edu.tw (T.-L.C.); 4 Department of Laboratory Medicine, National Taiwan University Hospital, Taipei 100225, Taiwan; 5 Institute of Bioinformatics and Structural Biology, College of Life Science, National Tsing Hua University, Hsinchu 300013, Taiwan; vamos0527@gmail.com; 6 Graduate Institute of Biomedical Electronics and Bioinformatics, National Taiwan University, Taipei 106319, Taiwan; andrewneteye4343@gmail.com (C.-H.H.); yukijuan@ntu.edu.tw (H.-F.J.); 7 Department of Life Science, National Taiwan University, Taipei 106319, Taiwan; 8 Institute of Biotechnology and Pharmaceutical Research, National Health Research Institutes, Miaoli 350401, Taiwan; alexhsieh@gate.sinica.edu.tw; 9 Department of Chemistry, National Tsing Hua University, Hsinchu 300013, Taiwan; 10 Biomedical Translation Research Center, Academia Sinica, Taipei 115202, Taiwan; 11 Department of Medical Science, National Tsing Hua University, Hsinchu 300013, Taiwan

**Keywords:** COVID-19, SARS-CoV-2, viral entry, receptor-binding domain, entry inhibitor, ACE2

## Abstract

Entry inhibitors against severe acute respiratory syndrome coronavirus 2 (SARS-CoV-2) are urgently needed to control the outbreak of coronavirus disease 2019 (COVID-19). This study developed a robust and straightforward assay that detected the molecular interaction between the receptor-binding domain (RBD) of viral spike protein and the angiotensin-converting enzyme 2 (ACE2) receptor in just 10 min. A drug library of 1068 approved compounds was used to screen for SARS-CoV2 entry inhibition, and 9 active drugs were identified as specific pseudovirus entry inhibitors. A plaque reduction neutralization test using authentic SARS-CoV-2 virus in Vero E6 cells confirmed that 2 of these drugs (Etravirine and Dolutegravir) significantly inhibited the infection of SARS-CoV-2. With molecular docking, we showed that both Etravirine and Dolutegravir are preferentially bound to primary ACE2-interacting residues on the RBD domain, implying that these two drug blocks may prohibit the viral attachment of SARS-CoV-2. We compared the neutralizing activities of these entry inhibitors against different pseudoviruses carrying spike proteins from alpha, beta, gamma, and delta variants. Both Etravirine and Dolutegravir showed similar neutralizing activities against different variants, with EC50 values between 4.5 to 5.8 nM for Etravirine and 10.2 to 22.9 nM for Dolutegravir. These data implied that Etravirine and Dolutegravir may serve as general spike inhibitors against dominant viral variants of SARS-CoV-2.

## 1. Introduction

The novel coronavirus disease (COVID-19) is an ongoing pandemic caused by severe acute respiratory syndrome coronavirus 2 (SARS-CoV-2). The disease outbreak started in November 2019 in China, and soon spread rapidly across countries. By March 2020, the World Health Organization (WHO) declared the COVID-19 pandemic. Since then, the COVID-19 pandemic has had devastating social and economic consequences around the globe.

SARS-CoV-2 uses the same cell entry receptor as SARS-CoV, the angiotensin-converting enzyme 2 (ACE2) [1,2]. The crystal structure of the receptor-binding domain (RBD) of viral spike protein in complex with ACE2 has been solved [3]. Compared with SARS-CoV, several residue changes in the RBD of SARS-CoV-2 spike stabilize two virus-binding hotspots at the RBD-ACE2 interface, explaining why SARS-CoV-2 RBD has a higher ACE2-binding affinity than that of SARS-CoV. Viral spike protein is critical for the viral entry into host cells. Specifically, a recombinant ACE2-Ig bound to SARS-CoV-2 RBD with a high affinity displayed a neutralizing effect of the SARS-CoV-2 spike-pseudotyped virus [4]. As the spike attachment to ACE2 is a critical step of viral infection, any agent that blocks viral attachment, such as neutralizing antibodies [5] or competitive entry inhibitors [6,7], can be applied to prevent viral infection.

Vaccination has proven successful in helping control the disease outbreak of COVID-19. However, viral variants may emerge with dangerous resistance to the immunity generated by the current vaccines to prevent COVID-19. Four viral variants named alpha, beta, gamma, and delta are currently defined as viral variants of concern, linked with either an increase in transmissibility or detrimental change in COVID-19 epidemiology, an increase in virulence or change in clinical disease presentation, or a decrease in the effectiveness of public health and social measures or available diagnostics, vaccines, and therapeutics [8]. The alpha variant (B1.1.7) identified in the United Kingdom was shown to have a substantial transmission advantage over other lineages, with at least 50% increased transmissibility [9,10]. The alpha variant was the predominant lineage between January and May 2021; then, it was replaced by the delta variant (B.1.617.2). The delta variant is characterized by the spike protein mutations T19R, Δ157-158, L452R, T478K, D614G, P681R, and D950N. Several of these mutations may affect immune responses directed towards the key antigenic regions of receptor-binding proteins (452 and 478) and the deletion of part of the N-terminal domain. Recent reports have indicated that the delta variant has exhibited a reduced sensitivity to certain monoclonal and polyclonal antibodies compared to the alpha variant [11]. After one dose, the effectiveness of the BNT162b2 and ChAdOx1 nCoV-19 vaccines against the delta variant was notably lower among persons with the delta variant than among those with the alpha variant [12]. On 26 November 2021, a new variant named Omicron (B.1.1.529) was designated as the fifth variant of concern (VOC) of the WHO, revealing that SARS-CoV-2 has the potential to develop beyond the available therapies [13].

To identify potential entry inhibitors against SARS-CoV-2 infection, we designed a quick and robust assay to measure spike attachment to the ACE2 receptor by applying a protein complementation technology of NanoLuc luciferase [14]. This assay allows the detection of RBD-ACE2 attachment in just 10 min. We applied this RBD-ACE2 attachment assay to screen entry inhibitors from 1068 FDA-approved drugs and validated the final candidates with viral neutralization assays using SARS-CoV-2 and spike-pseudotyped viruses of major variants. To comprehensively understand the binding patterns of Etravirine- and Dolutegravir-spike protein at an atomic scale, we believe that ab initio quantum chemical calculation, which has been utilized to clarify hydrogen bonding formations for years [15,16], will be an outstanding option in future work. This study identified Etravirine and Dolutegravir as promising viral entry inhibitors. We found that the neutralization efficiencies of Etravirine and Dolutegravir were not affected upon changes in key residues of viral RBD in major variants of SARS-CoV-2. These findings suggested that Etravirine and Dolutegravir may act as pan-viral entry inhibitors against predominant variants of SARS-CoV-2.

## 2. Results

### 2.1. RBD-ACE2 Attachment Assay

**Figure 1:**
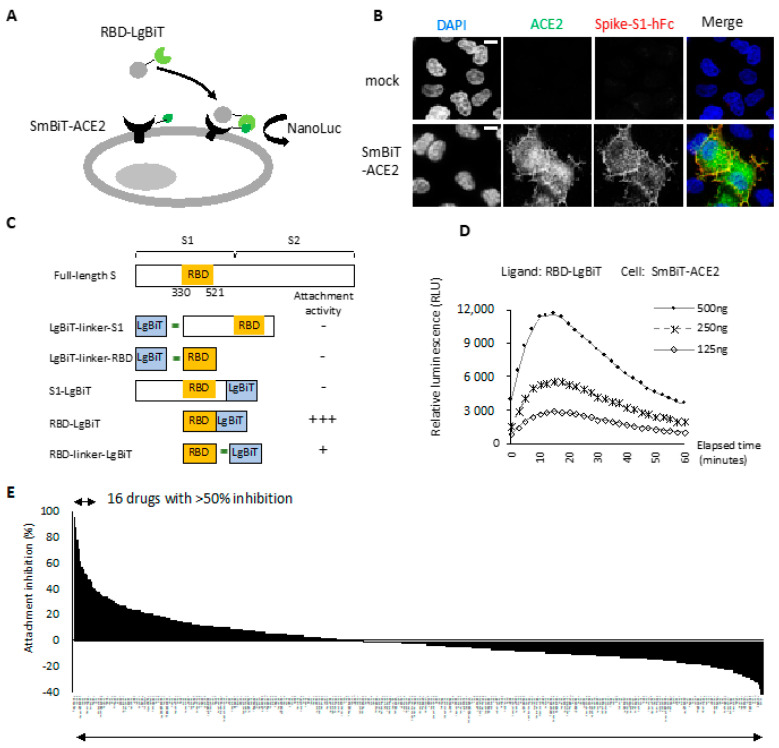
Identification of viral entry inhibitors by the cell-based RBD-ACE2 attachment assay. (**A**) Schematic of the RBD-ACE2 attachment assay. Reconstitution of NanoLuc occurs when recombinant RBD-LgBiT ligand attaches to the SmBiT-ACE2 on the cell surface of HeLa cells. RBD attachment activity is monitored by detecting bioluminescence signal following ligand treatment. (**B**) HeLa cells transfected with SmBiT-ACE2 were transiently incubated with recombinant S1-hFC. Successful attachment of S1-hFc was detected by immunostaining. Scale bar, 10 µm. (**C**) Schematic diagram of recombinant fusion proteins used as ligands for the RBD-ACE2 attachment assay. (**D**) RBD attachment activity measured using indicated amount of RBD-LgBiT as the ligand. All bioluminescence signals were recorded for one hour with 2 min interval time. Representative data of three independent experiments are shown. (**E**) Overall result of attachment inhibition using a single dose (20 μM) of FDA-approved compounds and pre-mixed with RBD-LgBiT before the incubation with SmBiT-ACE2 cells in the RBD-ACE2 attachment assay.

To monitor successful attachment between RBD and ACE2, we adopted the NanoLuc binary (NanoBiT) technology, which allows real-time assays to monitor the dynamics of protein–protein interactions in live cells [14,17]. The RBD-ACE2 attachment assay contained two major components: a stable cell line expressing ACE2 at the cell surface and a recombinant RBD protein with ACE2-binding activity (Figure 1A). We fused human ACE2 with the Small BiT (SmBiT) subunit of NanoLuc and transfected this combination into HeLa cells. In Figure 1B, we show that cell surface SmBiT-ACE2 was recognized specifically by a recombinant spike S1 protein. Next, we established a SmBiT-ACE2 stable expression cell line on HeLa cells with this expression construct. We then generated five different S1/RBD and LgBiT fusion constructs with codon optimization suitable for the mass production of recombinant proteins in bacteria (Figure 1C). Note that the RBD domain containing amino acids 330–521 of viral spike protein was used in this study [18]. We incubated these recombinant fusion proteins with SmBiT-ACE2-expressing cells and measured NanoLuc activity continuously for 1 h. No luminescence signal was detected with 500 ng of LgBiT-S1, LgBiT-RBD, and S1-LgBiT (Data not shown). In striking contrast, RBD-LgBiT induced a strong and robust luminescence signal upon incubation with SmBiT-ACE2-expressing cells (Figure 1D). RBD-linker-LgBiT that had an extra linker between RBD and LgBiT did not improve the luciferase activity (data not shown). As RBD-LgBiT gave the most robust NanoLuc activity in this assay, we used this recombinant protein in the following experiments.

We compared NanoLuc luciferase activity in the experimental setting with mock or SmBiT-ACE2 cells treated with 125, 250, and 500 ng of RBD-LgBiT. The luciferase activity increased with the amount of RBD-LgBiT ligand in the assay (Figure 1D). The peak value of luminance was detected at approximately 10 min after adding the substrate in the assay, followed by a slow signal decline. As such, we then defined the RBD-ACE2 attachment activity by measuring the peak luminescence signal detected at 10 min of reaction in subsequent studies.

### 2.2. Entry Inhibitor Screening from an FDA-Approved Drug Library

To identify novel viral entry inhibitors, we tested the inhibitory effects of an FDA-approved drug library on the RBD attachment, using 250 ng of RBD-LgBiT and 20 μM of a given drug in the initial screening. Notably, 16 out of 1068 drugs inhibited RBD attachment by over 50% (Figure 1E). The top 20 candidates with the highest attachment inhibitor activity were selected as subjects for a subsequent negative screening using the HiBiT-NanoLuc assay [19]. Among these candidates, 11 drugs inhibited HiBiT NanoLuc, and thus were excluded from the candidate list (Table 1). The final 9 candidates included Dolutegravir sodium, Etravirine, Gramicidin, ABT-199, Miconazole, Miconazole nitrate, Ospemifene, Ivermectin, and Aripiprazole. All these drugs displayed a dose-dependent effect on the inhibition of RBD attachment (Table 1, comparing the inhibitory effect between 100 μM and 20 μM). Dolutegravir, Etravirine, and Gramicidin were the most potent candidates that inhibited RBD attachment by more than 70% at a concentration of 20 μM.

**Figure 2:**
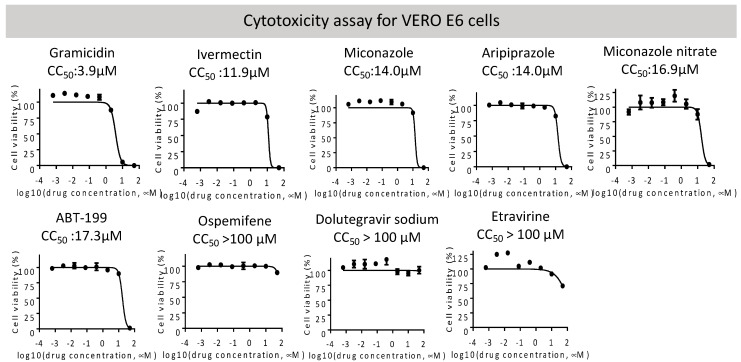
Overall cytotoxicity of indicated entry inhibitors was measured in VERO E6 cells by the CellTiter-Glo assay. CC50 values of each candidate are shown and listed in Table 2.

**Figure 3:**
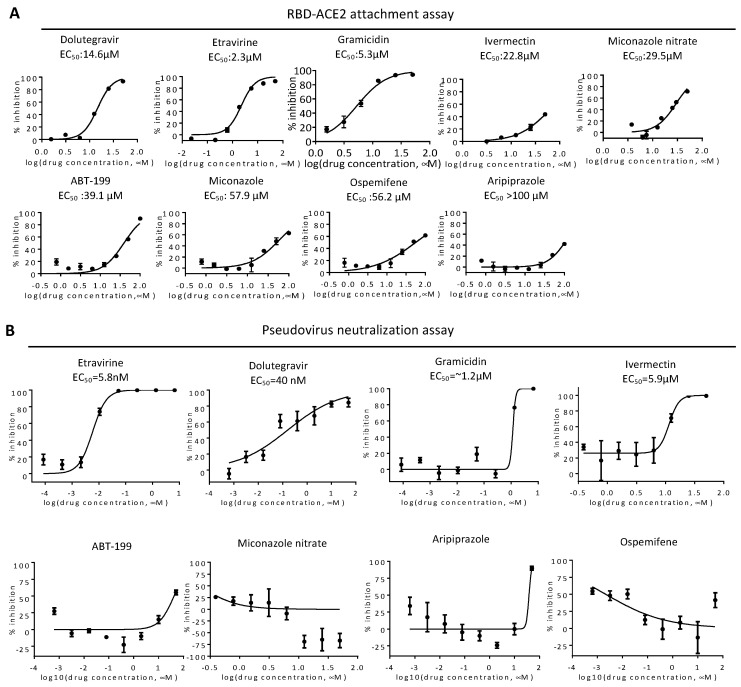
Validation of entry inhibitors by RBD-ACE2 attachment assay and pseudovirus neutralization assay. (**A**) EC50 of indicated drugs was determined by the RBD-ACE2 attachment assay. Mean and standard deviations from three technical replicates are shown. (**B**) HeLa cells expressing SmBiT-ACE2 were treated with entry inhibitors upon infection with spike-based pseudovirus carrying NanoLuc reporter genes. Representative results of pseudovirus neutralization activity of indicated entry inhibitors are shown. Inhibitor concentrations are presented in log scale for logarithmic interpolation. EC50 values of entry inhibitors in the neutralization assay are shown as indicated.

We next performed a cytotoxicity assay of these candidates on VeroE6 cells (Figure 2). Etravirine, Dolutegravir sodium, and Ospemifene were not toxic to VeroE6 cells, whereas other drugs showed various cytotoxicity with CC50 ranging from 3.9 μM for Gramicidin to 17.3 μM for ABT-199. These candidates were subjected to the RBD-ACE2 attachment assay (Figure 3). Among these candidates, Etravirine was most effective in blocking RBD attachment, with an EC50 of 2.3 μM. Gramicidin, Dolutegravir, and ABT-199 inhibited RBD attachment at EC50 values of 5.3, 14.6, and 39.1 μM, respectively. Miconazole and Miconazole nitrate inhibited RBD attachment at EC50 of 57.9 and 29.5 μM, respectively. Ivermectin was shown to inhibit the replication of SARS-CoV-2 in cell cultures via inhibiting the host importin alpha/beta-1 nuclear transport proteins, which are part of a critical intracellular transport process that viruses hijack to enhance infection [20]. We found that Ivermectin carried a weak entry inhibitor activity with an EC50 of 22.8 μM (Figure 3).

### 2.3. Neutralization of Spike Pseudovirus and SARS-CoV-2

To test neutralization activity, we produced a spike-based pseudovirus carrying a NanoLuc reporter gene (Figure 3B). We treated SmBiT-ACE2 cells simultaneously with the pseudovirus and entry inhibitors for 6 h, removed the virus, then incubated for an additional 16 h before bioluminescence detection. Etravirine and Dolutegravir potentially neutralized infections of spike pseudovirus, with EC50 values of 5.8 and 40 nM, respectively. On the other hand, the EC50 values of Gramicidin and Ivermectin were 1.2, and 5.9 μM. As CC50 values of Gramicidin and Ivermectin were 3.9 μM and 11.9 μM, we suspected that EC50 values measured in the neutralization assay with these two drugs might be affected by cytotoxicity (Figure 2). We therefore excluded Gramicidin and Ivermectin in the following assays.

**Figure 4:**
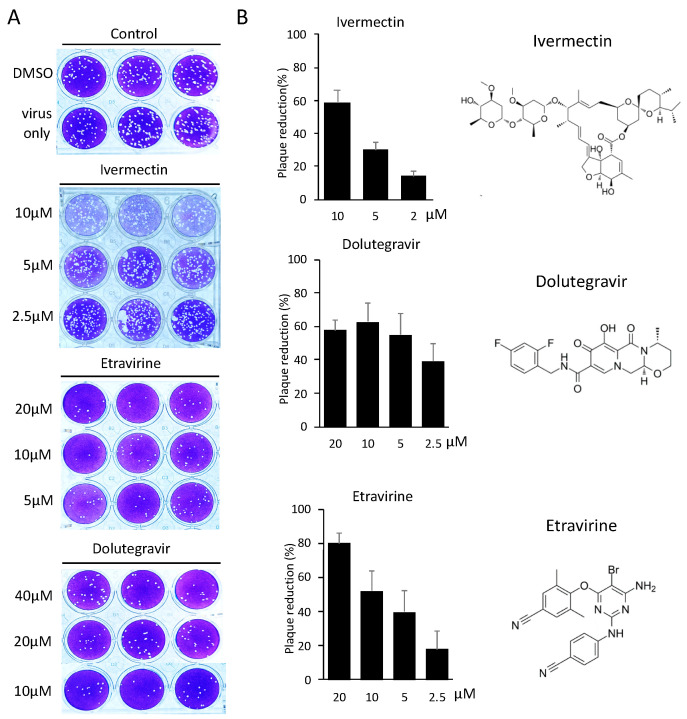
Neutralization efficiency of entry inhibitors was determined by SARS-CoV-2 PRNT. (**A**) Representative images of SARS-CoV-2 PRNT performed with selected entry inhibitors. Specifically, SARS-CoV-2 was pre-incubated with entry inhibitors for one hour, followed by one-hour infection with susceptible VeroE6 cells. Plaques forming were measured 5 days post-infection. (**B**) Neutralization efficiencies of entry inhibitors were determined by quantifying plaque numbers and displayed as percentage of plaque reduction. Chemical structures of entry inhibitors are shown on the right panel.

We next measured the neutralization efficiency of these inhibitors against SARS-CoV-2, using a conventional plaque reduction neutralization test (PRNT) [21]. We pre-treated entry inhibitors with SARS-CoV-2 for 1 h before infection with VERO E6 cells for another 1 h. Viral neutralization was measured by the quantitation of plaque numbers at 5 days post-infection (Figure 4A). Etravirine neutralized the infection of SARS-CoV-2 at an EC50 of 7.7 μM, and Dolutegravir at an EC50 of 4.2 μM (Figure 4B). We tested Ivermectin for viral neutralization as it was a known therapeutic candidate of COVID-19 [20]. Although the treatment of 10 μM Ivermectin blocked 60% of SARS-CoV-2 infection, we note that this result might be biased by the cytotoxicity (Figure 3), in addition to observing fainted cell staining in the plaque assay (Figure 4A). In short, these results indicated that Dolutegravir and Etravirine were safe entry inhibitors against SARS-CoV-2 as they can block viral entry at a dose with no detectable cytotoxicity. A summary result of these candidate viral entry inhibitors is provided in Table 2.

### 2.4. Etravirine and Dolutegravir Preferentially Interact with Spike RBD

**Figure 5:**
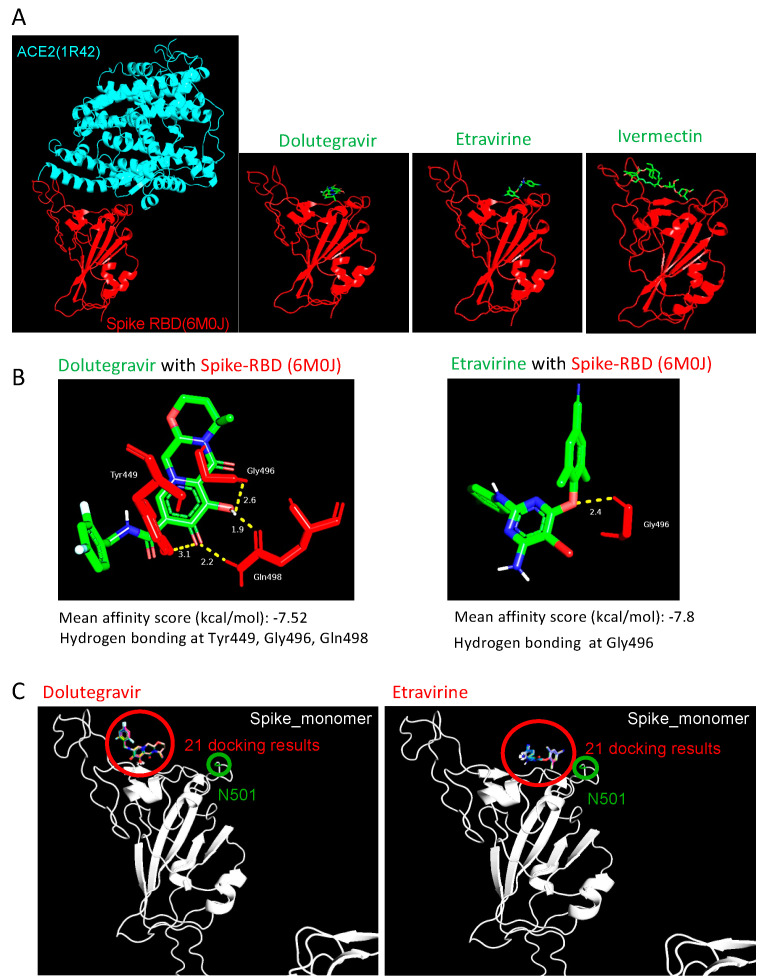
Structural simulation of Spike-RBD with drug candidates. (**A**) Visualization of the interaction pattern of ACE2-spike RBD (PDB ID: 6M0J). Dolutegravir and Etravirine structure were obtained from Pubchem; Ivermectin structure was obtained from ChemSpider. Potential interactions between entry inhibitors and ACE2 (PDB ID: 1R42) or spike RBD (PDB ID: 6M0J) were predicted by AutoDock Vina five times, and the mean affinity was further calculated. Interaction hydrogen bonds and residues were labeled by Pymol. (**B**) The predicted interaction of Dolutegravir (**left**) and Etravirine (**right**) with wild-type spike RBD. (**C**) Visualization of the 21 docking results of entry inhibitors (red circle) on the spike N501Y mutant (green circle). The N501Y residue located outside the binding interphases between Etravirine-RBD and Dolutegravir-RBD.

To determine the interactive nature of our entry inhibitors, we performed the molecular docking of Etravirine, Dolutegravir, and Ivermectin on both spike RBD and ACE2 by AutoDock Vina [22]. In the molecular docking results of ACE2, all predictive binding positions were not located at the RBD–interaction interphase, implying that ACE2 was not the primary target of these entry inhibitors (data not shown). On the other hand, we reasonably considered that spike had the interaction priority, which was identified with the high affinity between spike RBD and entry inhibitors at the ACE2-spike interphase, indicating that these entry inhibitors block virus infection by direct binding to viral spike RBD (Figure 5A). We next focused on Etravirine and Dolutegravir in the study. The mean affinity score of Dolutegravir to spike RBD was −7.52 kcal/mol, with three potential instances of hydrogen bonding with RBD at Tyr449, Gly496, and Gln498. Etravirine had a mean affinity score of −7.8 kcal/mol with one potential hydrogen bonding at Gly496 (Figure 5B). These high affinity scores and predicted hydrogen bonds detected between drugs and spike RBD support the roles of Dolutegravir and Etravirine as direct RBD inhibitors.

### 2.5. Dolutegravir and Etravirine as Pan-Entry Inhibitors of Predominant Viral Variants

During our manuscript preparation, multiple COVID-19 variants continued to circulate globally. In the United Kingdom (UK), an alpha variant strain, also known as 501Y.V1 or B.1.1.7 lineage, emerged with an unusually large number of mutations [23]. In South Africa, another beta variant of SARS-CoV-2 (known as 501Y.V2 or B.1.351 lineage) emerged independently. This variant shares some mutations with the alpha variant. Specifically, both variants carry a mutation in the RBD domain at position 501, where amino acid asparagine (N) is replaced with tyrosine (Y). We performed the molecular docking of Dolutegravir and Etravirine, and repeated this 21 times, independently. In all docking results, the binding positions of Etravirine or Dolutegravir were not changed by the presence of N501Y (Figure 5C). Accordingly, the interaction between Dolutegravir and viral RBD was not affected by N501Y.

**Figure 6:**
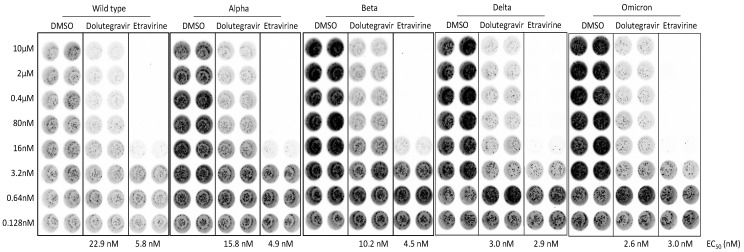
Neutralization activities of entry inhibitors against pseudoviruses carrying spike proteins of major SARS-CoV-2 variants. SmBiT-ACE2 cells were infected with SARS-CoV-2-pseudotyped viruses mixed with indicated DMSO, Dolutegravir, or Etravirine for 6 h and then removed from culture. Following 24 h post infection, bioluminescence signals of infected cells were detected by a bioluminescence imager and bioluminescent plate reader. Representative bioluminescence images of pseudotyped virus infection are shown. EC50 values of indicated entry drugs against different variants are shown.

We noted that the alpha variant was the predominant lineage between January and May 2021, which was then replaced by the delta variant (B.1.617.2). The delta variant is characterized by the spike protein mutations T19R, Δ157-158, L452R, T478K, D614G, P681R, and D950N. On 26 November 2021, a new variant named Omicron (B.1.1.529) was designated as the fifth variant of concern (VOC) of the WHO. The high number of mutations harbored on the spike protein make Omicron highly transmissible, less responsive to several of the currently used drugs, as well as potentially able to escape immune protection elicited by both vaccines and previous infection [13]. To explore whether the neutralizing activities of entry inhibitors have been lost in response to major clinical variants of SARS-CoV-2, we examined the neutralization activities of Dolutegravir and Etravirine against pseudoviruses carrying spike proteins of the alpha, beta, delta, and omicron variants. We infected SmBiT-ACE2 cells with these pseudoviruses and treated them simultaneously with various doses of Dolutegravir or Etravirine (Figure 6). By comparison of the neutralizing activities against wild type and other variants, we found that the EC50 values of Etravirine and Dolutegravir were not significantly changed among different viral variants. Dolutegravir neutralized wild type, alpha, beta, delta and omicron variants with EC50 values of 22.9, 15.8, 10.2, 3.0, and 2.6 nM, respectively. Etravirine neutralized wild type and different variants with EC50 values between 3 and 5.8 nM (Figure 6). Notably, both drugs showed the most effective entry inhibition against the omicron variant. Accordingly, we concluded that both Dolutegravir and Etravirine may act as pan-viral entry inhibitors for predominant variants of SARS-CoV-2.

## 3. Discussion

This study identified Etravirine and Dolutegravir as effective entry inhibitors of wild-type and predominant variants of SARS-CoV-2. We suggest Etravirine and Dolutegravir may serve as safe prophylactic agents of COVID-19. Interestingly, both drugs were initially designed for the treatment of human immunodeficiency virus (HIV). Etravirine is a second-generation non-nucleoside reverse transcriptase inhibitor (NNRTI), designed to be active against HIV with mutations that confer resistance to the two most commonly prescribed first-generation NNRTIs [24]. The crystal structure of HIV-1 reverse transcriptase and Etravirine were solved, and the conformation adaptability indicated that Etravirine is a potent inhibitor of wild-type and drug-resistant HIV variants [25]. In our RBD attachment assay, Etravirine inhibited RBD attachment at an EC_50_ of 2.3 µM. Strikingly, the EC_50_ of Etravirine in the pseudovirus neutralization assay was only 5.8 nM. As the spike pseudovirus was developed on the lentiviral backbone, we reasoned that Etravirine may inhibit both viral attachment and gene expression upon pseudoviral infection. This raises a potential caveat of identifying off-targeted drugs against lentivirus or HIV, if only pseudovirus neutralization assays can be used for drug screening against SARS-CoV-2. Accordingly, the RBD attachment assay can be applied as a backup assay.

Dolutegravir is an integrase inhibitor of HIV [26]. In 2018, the WHO recommended Dolutegravir as the preferred first-line and second-line HIV treatment in all populations, including pregnant women and those of childbearing potential. Compared with other antiretroviral drugs, Dolutegravir is effective, easier to take, and has a better side-effect profile, especially for patients who have had treatment failure [27]. These characteristics explained why Dolutegravir is wildly accepted in most countries for HIV therapy. In this study, Dolutegravir inhibited RBD attachment at an EC_50_ of 14.6 µM and pseudovirus neutralization at an EC_50_ of 40 nM. The low EC_50_ value detected for pseudovirus can be explained by the inhibition of lentiviral integrase, similar to the case of Etravirine. Notably, our molecular docking analysis revealed that Dolutegravir interacts with residues Try449, Gly496, and Gln498 of spike protein. As these three residues are key ACE2-interacting residues of spike protein [3], these data support Dolutegravir as a direct spike inhibitor to block the entry of SARS-CoV-2.

Etravirine and Dolutegravir were identified as inhibitors against main protease/mPro and RNA-dependent RNA polymerase/RdRP, respectively, in an independent molecular docking study [28]. In addition, Dolutegravir was proposed as a lead candidate of viral 3C-like protease/3CLpro inhibitor in a computational analysis [29]. These in silico analyses indicated that Etravirine and Dolutegravir may have beneficial off-target effects on SARS-CoV-2 infection. It will be important to explore the disease progression of hospitalized COVID-19 patients with HIV co-infection. In a large retrospective cohort study conducted on behalf of NHS England involving 17,282,905 registered adults, people living with HIV had a higher risk of COVID-19 death than those without HIV, after adjusting for age and gender, with hazard ratio of 2.90 [30]. As both entry inhibitors are usually taken in combination with other anti-HIV drugs, extracting individual drug information and clinical outcomes in SARS-CoV-2 and HIV co-infected patients may support the use of Etravirine and Dolutegravir for COVID-19.

Finally, the RBD-ACE2 attachment assay developed in this study is a rapid and robust method that detects RBD attachment in just 10 min. This assay does not require a BSL-2 laboratory facility and thus can be wildly applied to identify novel entry inhibitors or monitor neutralizing activities of antibodies or serum prepared from either convalescent plasma or experimental animals. We suggest this RBD attachment assay can be used to quickly detect protective neutralizing antibodies in COVID-19 patients.

## 4. Materials and Methods

### 4.1. Molecular Cloning and Cell Culture

SARS-CoV-2 S gene (original and *E. coli* codon-optimized) sequences were acquired from GenScript. Human ACE2 coding gene was obtained from Addgene (Plasmid #1786). To produce a recombinant Spike-RBD-LgBiT ligand, a codon-optimized Spike-RBD sequence was cloned into the pET28a expression vector through NcoI and XhoI, Ala-Gly-LgBiT (coding sequence from Promega, Madison, WI, USA); the coding sequence was then incorporated through XhoI (remaining in both ends). To ectopically express SmBiT-hACE2 in mammalian cells, a full-length hACE2 gene was subcloned into an EF-1α promoter-driven mammalian expression vector (which flanked with PiggyBac transposon inverted repeat sequence), and SmBiT (VTGYRLFEEIL from Promega)-Ala-Gly-Ala was used as a site-directed insertion between the hACE2 amino acid 17th and 18th residues. To utilize luciferase as a reporter in the pseudovirus assay, the Nluc (NanoLuc, from Promega)-Gly-Ser-Gly-T2A sequence was amplified and seamlessly cloned into the upstream of the RFP coding sequence in a lentiviral vector (pLAS2w.RFP-C.Ppuro, acquired from the RNAicore, Academia Sinica, Taiwan) by In-Fusion cloning (Takara Bio Inc., Kusatsu, Shiga, Japan). All cell lines involved in this research were regularly maintained in DMEM complete medium containing 10% FBS and Penicillin-Streptomycin solution and incubated in a 37 °C humidified incubator with 5% CO_2_. To generate SmBiT-hACE2-expressing cells, HeLa-Kyoto cells were co-transfected with plasmids containing the SmBiT-hACE2 construct mentioned above and PiggyBac transposase. After 48 h of incubation, transfected cells were put under hygromycin selection for one week and split into 96-well plates in order to obtain single-cell clones. Single-cell clones were then expanded and hACE2 expression was confirmed by immunofluorescence staining.

### 4.2. Indirect Immunofluorescence Staining

For examining SmBiT-hACE2 expression in single clones in addition to their binding capability with SARS-CoV-2-Spike protein, SmBiT-hACE2-expressing cells were fixed with 4% formaldehyde for 10 min, and incubated with anti-ACE2 antibody (Novus SN0754 clone, 1:500 dilution) and CoV-2-Spike-S1-hFc recombinant protein (Sino Biologicals, 120 ng per coverslip sample) for 1 h at room temperature. Anti-rabbit Alexa Fluor 594 and anti-human Alexa Fluor 488 secondary antibodies were then labeled for imaging by fluorescent microscopy. Images were taken by using Leica DMI6000 microscope with an HCX PL FL 63x/1.4 NA oil objective lens and Andor Neo sCMOS camera, which were all processed by MetaMorph software (Molecular Devices, LLC. San Jose, CA, USA).

### 4.3. Recombinant RBD Fusion Protein

Recombinant S1 and RBD fusion proteins were expressed in *E. coli* (Rosetta 2, Novagen) at 25 °C with IPTG induction for at least 3 h. After sonication and centrifugation, the supernatant of the cell lysate was discarded, and the pellet (inclusion body) was dissolved in the LB0304 buffer (SMOBIO, Inc., Hsinchu, Taiwan) containing urea as a denaturing reagent. The clear lysate containing denatured RBD-LgBiT proteins (or similar RBD-fusion constructs) was obtained by centrifugation, and then His-tag affinity chromatography with imidazole as elutant was conducted to purify the target protein. Eluates containing a high concentration of RBD fusion proteins were then precipitated with IPA and re-dissolved in LB0304 buffer, followed by gradual dilution with Tris-based renaturing buffer RB4020 (SMOBIO, Inc., Hsinchu, Taiwan) until at least 10-fold dilution was reached. If necessary, the target protein was further concentrated by ultrafiltration. The renatured protein was further analyzed by SDS-PAGE (SMOBIO, Inc., Hsinchu, Taiwan) for the estimation of protein concentration.

### 4.4. RBD-ACE2 Attachment Assay

SmBiT-hACE2 cells were seeded into 96-well white plates and incubated overnight prior to the attachment assay. For each assay, culture medium was removed and replaced with 50 μL of Opti-MEM I medium containing 250 ng of RBD-LgBiT protein, indicated protein competitors, or tested drugs. For measuring NanoLuc activity, a 20 μL of Nano-Glo live cell assay substrate (Promega) mixture (10 μL Opti-MEM, 9.5 μL diluent, 0.5 μL substrate) was added into each cell well. The luminescence signal was recorded immediately by the luminescent microplate reader (BioTek Synergy HTX) at 37 °C with a time-lapsed kinetics program of 2 min intervals for 1 h. For calculating the inhibition of all agents, luminescent data from the time point showing the highest signal in the negative control sample were chosen for downstream calculation. The percentage of attachment inhibition (%) was calculated by 1 − (luminescence signal of test sample)/(luminescence signal of negative control sample)) × 100. For the competition assay, 250 ng RBD-LgBiT was pre-mixed with different amounts of protein competitors in a 50 μL reaction volume for 15 min at 37 °C before the addition of Nano-Glo live cell substrate. Recombinant proteins of RBD-His and Spike S1-hFc were purchased from Sino Biological (Beijing, China). Full-length spike protein was a kind gift from Dr. Che Ma and Dr. Shang-Te Danny Hsu (Academia Sinica, Taipei, Taiwan). For FDA-approved drug library (TargetMol, L4200) screening, each compound was diluted, mixed and pre-incubated with 250 ng RBD-LgBiT ligand in Opti-MEM I medium for 15 min, and then added into SmBiT-hACE2-expressing cells.

### 4.5. Pseudovirus Neutralization Assay

For producing lentivirus-based pseudovirus, 5 μg transfer plasmid (pLAS2w.Nluc-T2A-RFP-C.Ppuro), 4 μg packaging plasmid (pCMVdeltaR8.91 from RNAicore, Academia Sinica, Taipei, Taiwan), and 1 μg spike-expressing plasmid (wild type, alpha, beta, and delta derivatives of pcDNA3.1-2019-nCoV-S-d18, kindly provided by the RNAicore, Academia Sinica, Taiwan) were co-transfected with Lenti-X 293T cells in a 10 cm culture dish. After being given aspiring medium the next day, the cells were fed with complete medium supplemented with 1% bovine serum albumin (BSA) for the following days. Supernatants at 30 and 60 h post-transfection were collected and stored as the virus stock. For each neutralization assay, SmBiT-hACE2 cells were cultured in 96-well white plate at the density of 3 × 10^5^ cells per well, one day before viral infection. Cells were simultaneously infected with pseudoviruses and treated with DMSO or indicated drugs for 6 h. Infected cells were washed three times with phosphate-buffered saline and incubated for an additional 18 h. Nano-GLo live-cell assay was used for measuring intracellular NanoLuc luciferase activity (Promega). The luminescence signal was recorded immediately by the luminescent microplate reader (BioTek Synergy HTX, Agilent Technologies Taiwan, Taoyuan, Taiwan) at 37 °C with a time-lapsed kinetics program of 2 min intervals for 1 h. For calculating the percentage of neutralization, luminescent data from the time point showing highest signal in negative control sample were chosen for downstream calculation. Percentage of neutralization (%) was calculated by 1 − (luminescence signal of the test sample)/(luminescence signal of negative control sample)) × 100.

### 4.6. SARS-CoV-2 Plaque Reduction Neutralization Test

Vero E6 cells were seeded in a 24-well culture plate in DMEM with 10% FBS and antibiotics one day before infection. SARS-CoV-2 virus (50−100 pfu) was incubated with compounds for 1 h at 37 °C, then added to the VeroE6 cells for another 1 h incubation. After removal of virus inoculum, the cells were washed once with PBS and overlaid with 1 mL overlay medium containing 1% methylcellulose for 5 days. For plaque staining, the cells were fixed with 10% formalin overnight and then stained with 0.5% crystal violet. The percentage of inhibition was calculated as 1-(VD/VC), where VD and VC refer to the virus titer in the presence and absence of the inhibitors, respectively. The minimal concentrations of the compounds required to reduce the plaque numbers by 50% (EC50) were calculated by regression analysis of the dose–response curves generated from the plaque assays.

### 4.7. Molecular Docking

To predict the interaction characteristics of the entry inhibitors with target proteins, AutoDock Vina [22] was applied as a molecular docking tool. Dolutegravir and Etravirine compound structure-data files (.sdf) were downloaded from Pubchem database (https://pubchem.ncbi.nlm.nih.gov/ (accessed on 16 September 2020)), and Ivermectin. sdf was downloaded from ChemSpider database (http://www.chemspider.com/ (accessed on 16 September 2020)). To convert the sdf file to a pdbqt file, we used OpenBabel-2.4.1 [31] and AutoDockTools-1.5.6 [32]. ACE2 (PDB ID: 1R42) [33] structure was obtained from Protein Data Bank database (https://www.rcsb.org/ (accessed on 16 September 2020)). The COVID-19 spike RBD domain (PDB ID: 6M0J) [34] structure was obtained through SWISS-MODEL (https://swissmodel.expasy.org/ (accessed on 16 September 2020)). Before performing molecule docking, these protein structures were also converted to pdbqt format by AutoDockTools. During ACE2 docking, the parameters were set as —center_x 58.67, —center_y 54.92, —center_z 25.751, —size_x 80, —size_y 80, —size_z 80, and exhaustiveness as 48. For spike RBD docking, we determined the detail parameters as —center_x -37.592, —center_y 32.34, —center_z 3.914, —size_x 28, —size_y 42, —size_z 20, and exhaustiveness as 48. After finishing each molecular docking, we chose the model with the strongest affinity as a candidate. For each compound, docking was operated for five times and calculated for mean affinity. Finally, the representative with the highest affinity score in these five candidates was displayed in Figure 6. To present the representative docking pattern, we applied Pymol (https://pymol.org/2/ (accessed on 19 September 2020)) software for hydrogen bond, distance between atoms, and interaction residue visualization.

### 4.8. Statistical Analysis

For calculating the attachment inhibition of all agents, luminescent data from the time point showing highest signal (approximately 10 min) in negative control sample were chosen for downstream calculation. Percentage of attachment inhibition was calculated by (1 − (luminescence signal of test sample)/(luminescence signal of negative control sample)) × 100. All quantitative data are presented as means ± SEM.

## 5. Conclusions

In summary, we developed a specific RBD-ACE2 attachment assay and identified Dolutegravir and Etravirine as effective and broad-spectrum entry inhibitors against major dominant variants of SARS-CoV2. As both drugs can be orally administrated, we suggest that these entry inhibitors can be used as pre- and post-exposure prophylactic treatments for COVID-19.

## Figures and Tables

**Table 1:** Top 20 hits of entry inhibitors in the initial screening.

No.	Hits	Positive Screening RBD Attachment Assay			Negative Screening HiBiT Assay ^†^		
		% Inhibition (100 μM)	% Inhibition (20 μM)	* Dose Response	% Inhibition (100 μM)	% Inhibition (20 μM)	INHIBIT NanoBiT ^‡^
1	Thonzonium Bromide	99	93	Yes	52	36	Yes
2	Dabrafenib (GSK2118436)	96	85	Yes	97	88	Yes
3	Dolutegravir sodium (GSK1349572)	96	82	Yes	62	1	No
4	Etravirine (TMC125)	90	78	Yes	31	0	No
5	Crystal Violet	99	73	Yes	86	17	Yes
6	Gramicidin	95	70	Yes	35	0	No
7	Clevidipine butyrate	93	69	Yes	93	79	Yes
8	Nitazoxanide	93	69	Yes	95	77	Yes
9	Nimodipine	96	64	Yes	97	79	Yes
10	Felodipine	96	63	Yes	99	88	Yes
11	Nicardipine hydrochloride	86	58	Yes	88	64	Yes
12	Aripiprazole	71	56	Yes	21	13	No
13	Phenazopyridine hydrochloride	91	52	Yes	83	48	Yes
14	Miconazole nitrate	83	52	Yes	12	3	No
15	ABT-199	96	51	Yes	43	0	No
16	Miconazole	79	50	Yes	15	6	No
17	Dronedarone hydrochloride	93	41	Yes	86	21	Yes
18	Atovaquone	95	39	Yes	96	54	Yes
19	Ospemifene	68	39	Yes	8	3	No
20	Ivermectin	68	29	Yes	4	0	No

* Positive screening was performed with the RBD attachment assay. Dose response is defined by the correlation between the dose of drug and the degree of inhibition in the RBD attachment assay. ^†^ Negative screening was performed by measuring NanoBiT activity upon the attachment of RBD-HiBiT to cells expressing SmBiT-ACE2. Inhibition of NanoBiT activity was determined by primarily picking up >70% inhibition in 100 μM, then including >35% inhibition in the presence of 20 μM drugs. ^‡^ Drugs with an inhibitory effect above 20% at 20 μM treatment concentration are likely genuine NanoBiT inhibitors as they strongly inhibited NanoBiT activity in the negative screening.

**Table 2:** Summary of candidate entry inhibitors identified in this study.

Name (CAS ID)	Major Target	Application			EC_50_ (μM)	
			VeroE6 CC_50_ (μM)	RBD Attachment	Pseudovirus Wild Type	SARS-CoV-2 PRNT
Dolutegravir sodium 1051375-19-9	HIV integrase	HIV integrase inhibitor	>100	14.6	0.04	4.2
Etravirine 269055-15-4	HIV reverse transcriptase	Non-nucleoside reverse transcriptase inhibitor (NNRTI) for HIV	>100	2.3	0.006	7.7
Miconazole nitrate 22832-87-7	Unknown	Anti-fungal agent	16.9	29.5	n.a.	n.a
Gramicidin 1405-97-6	MRP1	Anti-bacterial	3.9	5.3	1.2	n.a.
Ivermectin 70288-86-7	GluCl channel; P2X purinergic receptor	Broad-spectrum antiparasitic drug	11.9	22.8	5.9	n.a.

n.a. indicates not applicable.

## Data Availability

Not applicable.
